# *N*-acetyltransferase (*nat*) Is a Critical Conjunct of Photoperiodism between the Circadian System and Endocrine Axis in *Antheraea pernyi*

**DOI:** 10.1371/journal.pone.0092680

**Published:** 2014-03-25

**Authors:** Ahmed A. M. Mohamed, Qiushi Wang, Jadwiga Bembenek, Naoyuki Ichihara, Susumu Hiragaki, Takeshi Suzuki, Makio Takeda

**Affiliations:** 1 Graduate School of Agricultural Science, Kobe University, Kobe, Japan; 2 Graduate School of Science and Technology, Kobe University, Kobe, Japan; University of Texas Southwestern Medical Center, United States of America

## Abstract

Since its discovery in 1923, the biology of photoperiodism remains a mystery in many ways. We sought the link connecting the circadian system to an endocrine switch, using *Antheraea pernyi*. PER-, CLK- and CYC-ir were co-expressed in two pairs of dorsolateral neurons of the protocerebrum, suggesting that these are the circadian neurons that also express melatonin-, NAT- and HIOMT-ir. The results suggest that a melatonin pathway is present in the circadian neurons. Melatonin receptor (MT2 or MEL-1B-R)-ir in PTTH-ir neurons juxtaposing clock neurons suggests that melatonin gates PTTH release. RIA showed a melatonin rhythm with a peak four hours after lights off in adult brain both under LD16∶8 (LD) and LD12∶12 (SD), and both the peak and the baseline levels were higher under LD than SD, suggesting a photoperiodic influence. When pupae in diapause were exposed to 10 cycles of LD, or stored at 4°C for 4 months under constant darkness, an increase of NAT activity was observed when PTTH released ecdysone. DNA sequence upstream of *nat* contained E-boxes to which CYC/CLK could bind, and *nat* transcription was turned off by *clk* or *cyc* dsRNA. dsRNA^NAT^ caused dysfunction of photoperiodism. dsRNA^PER^ upregulated *nat* transcription as anticipated, based on findings in the *Drosophila melanogaster* circadian system. Transcription of *nat, cyc* and *clk* peaked at ZT12. RIA showed that dsRNA^NAT^ decreased melatonin while dsRNA^PER^ increased melatonin. Thus *nat,* a clock controlled gene, is the critical link between the circadian clock and endocrine switch. MT-binding may release PTTH, resulting in termination of diapause. This study thus examined all of the basic functional units from the clock: a photoperiodic counter as an accumulator of mRNA^NAT^, to endocrine switch for photoperiodism in *A. pernyi* showing this system is self-complete without additional device especially for photoperiodism.

## Introduction

The biological mechanisms of photoperiodism remain a big mystery since first recognized in 1923, mainly due to the complex nature of the system. The photoperiodic system is a black-box consisting of several functional subunits: 1) photoreceptors, 2) photoperiodic clock, 3) the photoperiodic counter, and 4) an endocrine switch for altering developmental fate, metamorphosis and metabolism in insects [1].

The Chinese Tasar Moth, *Antheraea pernyi* undergoes facultative diapause at pupal stage; dormancy is induced by short days, while development continues under long-day conditions. Choice of either of these phenotypes is regulated by the release or suppression of release of the prothoracicotropic hormone (PTTH).

In this moth, PER-ir cells have been found in three groups of dorsolateral neurosecretory cells of the brain [2]; although PER protein and *per* mRNA levels oscillated in these cells, extensive migration of PER to the nucleus was not observed as decisively as in *D. melanogaster.* There is no delay in timing of the peak expressions between *per* mRNA and PER protein, unlike in *D. melanogaster* where massive nuclear translocation of the PER/TIM heterodimer occur*s* at particular ZTs [3]. A rhythm in PER expression in *A. pernyi* is accompanied by a rhythm in the expression of antisense *per* mRNA with an antiphase relationship. The same patterns have been observed in other insects, *Rhodnius prolixus* [4] and *Bombyx mori* [5]. In *Periplaneta americana*, PER-ir is not only distributed in the focused locus of the optic lobe [6] but also in both the dorso-lateral region of the protocerebrum and the pars intercerebralis (PI) [7]. Matsui et al., [8] characterized a circadian clock element in the PI in *P. americana*; surgical extirpation of the PI induced arrhythmicity in locomotor activity in constant darkness (DD).

Most investigators consider that photoperiodism is a special mode of circadian system function, although a concrete mechanism has not been identified, at least at the molecular level. Therefore, knowledge on the molecular architecture of circadian systems in *D. melanogaster* could provide accessibility to the molecular mechanisms of the photoperiodic system, since the most well-documented circadian pacemaker is that of *D. melanogaster*. This clock, which regulates adult locomotor activity and eclosion, operates based on interlocked negative transcription/translation feedback loops [9]–[12]. In this system, several clock genes and their proteins are involved in the interlocked feedback loops. In each loop, positive elements control the transcription of the negative elements. Briefly, the transcriptional activators CLOCK (CLK) and CYCLE (CYC) form a heterodimer that binds to E-box sequences on the promoter regions of *period* (*per*) and *timeless* (*tim*), triggering their transcription [9]. After translation of the mRNA of the two key genes *per* and *tim*, their proteins PERIOD (PER) and TIMELESS (TIM) accumulate throughout the dark phase of the daily cycle. As protein, they form a heterodimer PER/TIM in the cytoplasm and interact with several phosphatases and kinases that regulate timing of accumulation and nuclear entry, as well as the stability of PER/TIM and their ability to dimerize. PER/TIM dimers act as negative regulators of the CYCLE/CLOCK (CYC/CLK) heterodimer [13]. CLK, CYC, and PER have an important similarity in their sequences in that they have PAS domains, which function as dimerization domains [14]. Kinases such as DOUBLETIME (DBT), SHAGGY (SGG) and CASEIN KINASE II (CKII), and phosphatases, such as protein phosphatases 1 (PP1) and 2A (PP2A), phosphorylate/dephosphorylate PER or TIM and also CLK inside the nucleus [15]–[24]. TIM alone is not a sufficient repressor for CLK/CYC, while PER alone can act as an effective repressor [25].

CRYPTOCHROME (CRY), a homologue to DNA photolyase, is an important circadian clock regulator. In *D. melanogaster*, its mode of action is to control the degradation of TIM through JETLAG (JET) [26], [27], after activation by blue light. TIM degradation exposes PER to the kinase DBT that phosphorylates PER, affecting the stability of PER and the feedback loop [28]. The other possible function of CRY is to mediate cross-talk among clock cells. CRY is a dimerization partner with PER in mammalian PER negative feedback system and Lepidoptera has two CRY types, the *Drosophila* type and a mammalian type [29]. *Apis mellifera* has only the mammalian type [30].

A link between the circadian and photoperiodic systems remains unclear in insects, but they have been more extensively studied in plants and mammals [31]–[34]. However, there are some evidence suggesting the connection between the circadian clock system and diapause. For example, the mutation in the promoter region of *timeless* in the Drosophilid fly, *Chymomyza costata*, is responsible for the inability of the mutant strain to diapause [35], [36]. Similary, in *Drosophila triauraria*, allelic differences in two circadian clock genes (*timeless* and *cytochrome*) in different strains affected differently on the incidence of diapause [37]. Han and Denlinger [38] showed that length variation in a specific region of the circadian clock gene *period* correlated in different ways on the incidence of pupal diapause in *Sarcophaga bullata*. Here, we focus on the pathways linking the circadian clock with the photoperiodic response. We propose that the link should reside where circadian controlled genes (ccg) regulate photoperiodic response via neuroendocrine mechanism. Our primary target is the indolamine pathway. Melatonin may stimulate the release of a critical neurohormone, PTTH. Melatonin production is dependent upon the enzyme arylalkylamine *N*-acetyltransferase (aaNAT). *aanat* may thus be the critical ccg in this system. This hypothesis is examined in this report by a series of RNAi experiments, targeting *aanat*, two transcription regulators, *clk* and *cyc*, and a negative regulator, *per*.

RNAi is an effective technique for disrupting expression and thus function of individual genes that have been otherwise nearly impossible to analyze [39]. In this report we have successfully used RNAi technique to demonstrate that in *A. pernyi* the molecular mechanism of the circadian system controlling photoperiodism is similar to the mechanisms controlling circadian rhythms.

## Materials and Methods

### Ethics Statement

This study was carried out in strict accordance with the recommendations in the Guide for the Care and Use of Laboratory Animals of Kobe University. The protocol was approved by the Committee on the Ethics of the Animal Experiments of Kobe University (Permit Number: 19-5-01). All surgery on rabbits was performed under sodium pentobarbital anesthesia. The moth is industrially produced in China for textile and food purposes and small scale in Japan. Therefore no permission is required and there is no ecological risk to local biodiversity; the only reason we bought this stock was to ensure uniformity of diapause.

### Insects

Wild-grown cocoons containing pupae in diaupase and of a univoltine strain of *A. pernyi* were harvested in He Nang Province, PRC, in October and either shipped or personally carried by researchers to Japan. The strain was shipped or carried from ShengYan, Liaoning Province, to Japan in October. The cocoons were stored under LD 12∶12 at 25°C for 2 weeks, during which non-diapause pupae or diapause pupae that resumed development during transportation and handling emerged as adults. For the remaining pupae (>95%), diapause was maintained under LD 12∶12 at either 25°C or 5°C. Diapause pupae were used for physiological experiments within 4 months, during which photoperiodism was securely maintained. Dissection for the brain-subesophageal ganglion complex (Br-SOG) for the experiments was conducted during the daytime after activating the diapause pupae 5 days under long day condition (LD 16∶8), unless otherwise mentioned.

### Production of antisera against *Pa* PER, *Dm* aaNAT, *Ap* aaNAT, *Bm* CYC and *Bm* CLK

We raised antibodies against Pa (*Periplaneta americana*) PER, *Ap* (*A. pernyi*) CYC, *Ap* CLK, *Dm* (*Drosophila melanogaster*) NAT1 and *Ap* NAT. The antigens were produced as GST- or MBT-fusion proteins. Specifically, for construction of the GST-*Periplaneta* PERIOD fusion protein, cDNA encoding *Periplaneta period* was inserted in pBluescript SK vector provided by Dr. Steven M. Reppert. This cDNA was digested using EcoRI and XhoI. Digested cDNA was separated using agarose gel electrophoresis and an approx. 1000 bp band was recovered using Ultra Clean 15 kit (MO Bio). By using DNA ligation Kit Ver. 2, the digested, purified cDNA was ligated to the pGEX 5X-1 that was also digested using EcoRI and XhoI. *E. coli* was transformed using the ligation product of this reaction. A transformed colony was selected and cultured in 4 ml of LB medium at 37°C for 12 hours. Then, the culture was added to LB medium and incubated at 37°C for 4 hours. One ml of 1 M IPTG was added to this medium to induce protein synthesis of the fusion protein, which was further incubated at 30°C for 1 hour. Then, the medium was centrifuged at 3000 rpm for 20 minutes and the precipitate was collected. The precipitate was suspended in 20 ml of Tris-HCl buffer (20 mM pH 8.0 Tris-HCl, 30 mM NaCl, 10 mM EDTA, 2 mM PMSF) and the destruction of the cell wall was carried out by a series of freeze-thawing. The sample was supplemented with 1.2 ml of 10% Triton X-100 and 480 μl of 5 M NaCl. Then, sonication was carried out using SONIFIER 250 (Branson) for 20 min. The sonicated sample was centrifuged at 10,000 rpm for 30 min. Then, precipitate was collected and suspended into 50 ml of 1 M sucrose. The sample was centrifuged at 8,000 rpm for 20 minutes and precipitate was suspended into 50 ml of 2% Triton X-100 in 10 mM EDTA buffer and incubated at 4°C for 12 hours. The incubated sample was dissolved into the SDS-PAGE sample buffer (25 mM, pH 6.8 Tris HCl, 2% SDS, 3.75% β-mercaptoethanol, 3.75% glycerol, 0.005% Bromo Phenol Blue) and SDS-PAGE was carried out using 10% acrylamide gel. Then, the band of GST-PER was cut out from the polyacrylamide gel. The band was minced and was put into the dialysis tube containing SDS running buffer. The dialysis tube was soaked in SDS running buffer in the MUPID. To separate the fusion protein from the polyacrylamide gel matrix, 100 V was applied for 4 hours. Fusion protein including buffer was collected in the dialysis tube. The protein thus separated was dialyzed in distilled water for 12 hours.

For *Drosophila* aaNAT, we followed the same procedures as with *Periplaneta* PERIOD till we had the sonicated sample which was centrifuged at 10,000 rpm for 30 min. The supernatant was collected and loaded onto a 1 ml Glutathione Sepharose 4B (GE Healthcare Bioscience, Buckinghamshire, England) column equilibrated with the column buffer (pH 7.5, 20 mM Tris-HCl, 0.5 mM EDTA, 100 mM NaCl, 5 mM βmercaptoethanol, 0.01% NP-40). This column was washed with 30 ml of the same buffer and 10 ml of 50 mM Tris-HCl buffer, pH 8.0. The fusion protein was eluted with 3 ml of elution buffer (pH 8.0, 50 mM Tris-HCl, 20 mM glutathione). The eluted protein was dialyzed in 2 l of distilled water for 12 hours.

A cDNA containing the complete ORF of *Ap* aaNAT (261 residues) was obtained from the pupal brain of *A. pernyi* by standard techniques and subcloned into pGEX6p-1 (GE) in-frame downstream of the GST. Recombinant *Ap*NAT was induced in *E. coli* DH5α cells by adding 0.1 mM IPTG for 5 hrs at 37°C and purified on glutathione Sepharose 4B affinity chromatography columns followed by PreScission protease (GE Healthcare) digestion according to the manufacturer’s protocol. After digestion, the GST moiety and PreScission protease were absorbed in glutathione Sepharose 4B resin.

Recombinant CYC and CLK proteins were obtained by subcloning of cDNA corresponding to a 191-amino-acid region of *Bm*CYC (496 to 686 residues), and a 185-amino-acid region of *Bm*CLK (378th to 562nd), into pET22b plasmid (Novagene, Darmstad, Germany) in-frame and upstream of 6xHis. Both recombinant *Bm*CYC and BmCLK proteins were induced in BL21 (DE3) cells of *E. coli* by incubation in 0.1 mM IPTG for 3 h at 37°C. Recombinant proteins were purified on a HiTrap Chelating HP column (GE Healthcare) according to the manufacturer’s protocol. Following purification, both recombinant proteins were dialyzed against phosphate buffer (PB; 50 mM sodium phosphate pH 5.5) and trapped on a HiTrap Q HP column (GE Healthcare) to exclude imidazole completely.

### Immunization to Rabbit

Fifty micrograms of protein (*Pa*PER, *Dm*NAT, *Ap*NAT, *Bm*CYC and *Bm*CLK as GST-, MBT-hybrid or His-tag), solution in PBS and 1.2 volumes of Freund’s complete adjuvant were mixed until emulsified. This emulsion was injected subcutaneously into (New Zealand white female rabbits). The immunization was repeated. Three weeks later, 50 μg of protein solution in PBS and 1.2 volumes of incomplete Freund’s adjuvant were mixed to complete emulsification. After 1 week, the blood of each rabbit was collected and incubated at 37°C for 1 hour. Then, the blood was incubated at 4°C for 12 hours and centrifuged at 3,000 rpm for 15 min. The supernatant was collected and used as the antiserum.

### Production of Other Antibodies and Anti-peptide Antibodies

Anti-rat liver HIOMT (monoclonal) and anti-*Ap*PTTH were gifts from the late Dr. Takeo Deguchi and Dr. Ivo Sauman, respectively. Anti-melatonin and anti-*h*MT2 sera were purchased [5], [40]. Specificities of other antibodies have been described elsewhere [2], and technical data provided by the manufacturer (Santa Cruz).

### Immunohistochemical Staining

Dissected brains were fixed in 50x volumes of Bouin solution for 2 hours, and then were washed 3 times in 80% ethanol for 15 min. Then, the brains were passed through 90% ethanol (15 min), 95% ethanol (15 min), 100% ethanol (15 min), 100% ethanol (30 minutes) and xylene (10 minutes, 2 times) sequentially for dehydration and dealcoholation. Tissues were embedded in paraffin and 8 μm sections were cut. The sections were stained based on an ABC method using the Vectastain elite ABC peroxidase kit (Vectastain, Vector Laboratories, Burlingame, CA, USA) following the manufacturer’s instructions. For deparaffinization and blocking, the sections were passed through xylene (30 minutes, twice), 100% ethanol (15 minutes, twice), 90% ethanol (15 minutes), 80% ethanol (15 minutes), 70% ethanol (15 minutes), 50% ethanol (15 minutes), water (5 minutes, twice), TBS-Tween (TBST, 10 minutes) and TBST-containing 1% BSA (1 hour), sequentially. The sections were incubated in the primary antiserum solutions (1,000x anti-*Pa*PER antiserum or 200x anti-*Ap*NAT antiserum diluted 1,000x with TBST containing 1% BSA) at 4°C, for 12 hours. After washing 3 times in TBST for 15 minutes, the sections were incubated in the biotinylated anti-rabbit IgG diluted 200x with TBST for 1 hour. After washing 3 times in TBST each for 15 minutes, the sections were incubated in the biotinylated peroxidase and avidin mixed solution for 30 minutes. After washing three times in TBST for 15 minutes each, the sections were stained with the DAB solution (pH 7.2, 0.1 M Tris-HCl, 0.1% DAB, 0.02% H_2_O_2_) for 5 minutes.

Colocalization of two antigens was conducted either on adjacent sections or by double labeling of identical sections (antibodies derived from different animals). *Ap*PTTH/*h*MT2 double labeling was conducted as follows: *Ap*PTTH antibody was diluted at 1∶2000, and *h*MT2 antibody at 1∶800. Drop cocktail of both primary antibodies (anti-*Ap*PTTH and anti-*h*MT2) diluted in TBST containing 1% BSA was used to incubate the sections overnight at 4°C. After rinsing (3×) with TBST, the slides were incubated with horse anti-goat IgG (H+L)-biotin (Vector Laboratories) for 1.5 h. After rinsing (3×) with TBST, the slides were incubated with Alexa Fluor 488-conjugated (green) goat anti-rabbit IgG for 60 minutes at rT (Invitrogen, Tokyo, Japan). After washing in TBST, the biotin signal was visualized with red fluorophore using a TSA Labeling Kit #42 with Alexa Fluor 555 (Invitrogen, Tokyo, Japan). Finally, the slides were rinsed (3×) with TBST, mounted in Aqua Ploymount and observed using a BX50F4 microscope (Olympus, Japan).

For the double labeling (antibodies derived from the same animal), experiments were performed according to the method mentioned in Hiragaki [41]. For example, anti-*Bm*CYC and *Bm*CLK (rabbit) with anti-*Pa*PER (rabbit) and anti-*Bm*CLK (rabbit) with anti-*Ap*NAT (rabbit) were used as follows. Antibody 1 (Table 1) was diluted and the slides were incubated in the antibody solution overnight at 4°C. After rinsing (3×) with TBST, the slides were again incubated with the secondary antibody for 60 minutes. After rinsing (3×) with TBST, they were treated for 30 minutes with VECTASTAIN ABC reagent (Vectastain ABC KIT PK-6101). Then, sections were treated with TSA Biotin System (PerkinElmer), which can induce covalent bonds between tissue and biotin on the position of first color. After the photography was taken, antibody 1 was stripped out of the sections for 24 h at rT in stripping buffer (100 mM 2-mercaptoethanol, 50 mM glycine-HCl, pH2.2) in a 40 V horizontal electric field. The sections were then incubated with antibody 2 (Table 1) overnight at 4°C. The sections were then treated for 30 minutes with VECTASTAIN ABC reagent. After rinsing (3×) with TBST, the slides were incubated with Alexa Fluor 488-conjugated (green) goat anti-rabbit IgG for 60 minutes at rT. After rinsing (3×) with TBST, the biotin signal was visualized with green fluorophore using a TSA Labeling Kit #42. Finally, the slides were rinsed (3×) with TBST, mounted in Aqua Ploymount and observed using a BX50F4 microscope (Olympus, Japan).

**Table 1:** Summary of double-labeling (both primary antibodies from the rabbit) experiments.

Combination	Antibody 1	Dilution	Antibody 2	Dilution
*Bm*CYC+*Pa*PER	CYC	1∶600	PER	1∶1000
*Bm*CLK+*Pa*PER	CLK	1∶600	PER	1∶1000
*Ap*NAT+*Pa*PER	NAT	1∶1000	PER	1∶1000
*h*MT2+*Ap*PTTH	*h*MT2	1∶800	PTTH	1∶2000
*Ap*NAT+*Ap*PTTH	NAT	1∶1000	PTTH	1∶2000

We employed other antibodies based on antigens from species other than *A. pernyi* but effectively similar data were obtained.

Source: *Bm*CYC, *Pa*PER, *Bm*CLK and *Ap*NAT from M. Takeda, Kobe University, Japan: *Ap*PTTH from Sauman and Reppert, 1996: *h*MT2 purchased from Santa Cruz; sc-13174.

### Measurement of Brain Melatonin Content and *N*-acetyltransferse Activity

The brain-subesophageal ganglion complex (Br-SOG) was dissected from pupae in ice-cold PBS solution. Samples were immediately frozen and stored at −70°C until use. The samples were homogenized individually with 100 μl of chloroform, centrifuged and evaporated. Five hundred μl of assay buffer was added to dissolve the dried content. MEL content was measured by Radioimmunoassay (RIA) procedure. Anti-MEL serum and tritiated [^3^H]-MEL were purchased from Stockgrand Ltd. and Amersham International, respectively. Crystalline MEL was obtained from Sigma-Aldrich, St Louis, USA, with which a stock solution of 1 mg/ml MEL was prepared and stored at −20°C. The antiserum was stored at −80°C and diluted with assay buffer before use to give an initial dilution of 1∶600. Two hundred μl of tissue sample was added to a tube with an additional 100 μl of assay buffer, 100 μl of [^3^H]-MEL and 100 μl of diluted antibody. After incubation for 24 hours at 4°C, 500 μl of DCC (Dextran Coated Charcoal) was added to the reaction mixture, which was then incubated for 15 minutes at 4°C. This was centrifuged at 700×g for 15 minutes at 4°C and the supernatant was added to vials containing 3.6 ml of scintillation cocktail (Atomlight, Packard). Radioactivity was then measured using a scintillation counter (Aloca, LSC-3500).

Enzymatic activity of NAT was measured based on the radioenzymatic assay. Briefly, stored, frozen (Br-SOGs) those were dissected out at ZT6 and ZT18 were homogenized using a homogenizer in ice-cold buffer. Samples were centrifuged and the supernatant was used as a crude enzyme solution. NAT activity was measured by counting radioactivity of *N*-acetyltryptamine formed from tryptamine, and [^14^C]-acetyl-CoA [40], [41]. 0.1 M citric acid-Na_2_NPO_4_ (pH 5.0–6.0), 0.1 M Clark-Lubs KH_2_PO_4_-NaOH (pH 6.0–8.0) and 0.1 M Clark-Lubs H_3_BO_3_-KCl-NaOH (pH 8.0–10.0) were used to construct a pH curve. Samples were incubated at 37°C for 15 minutes. 100 μl of 5% acetic acid was added to the sample to stop the reaction, and then one ml of scintillation cocktail was added and the tube was vortexed for one minute. Liquid scintillation cocktail consisted of toluene:isoamylalcohol (97∶3) containing 4 g of PPO, and 0.1 g of POPOP in one liter of solution. The plastic tube was placed in a glass vial and radioactivity was measured using a scintillation counter (Aloca, LSC-3500).

### Protein Measurement

Protein determination for radioimmunoassay was performed according to Bradford [42] with BSA as standard (Sigma-Aldrich, St Louis, USA). Optic density (OD 595 nm) of the mixture versus reagent blank was measured using an Epoll 20 spectrophotometer. The values are given as the mean of duplicate determinations.

### Inverse PCR for Amplification of Upstream Regulatory Region

Genomic DNA was isolated from the brains of the pupae using Mammalian Genomic DNA kit according to the manufacturer’s instructions (Sigma-Aldrich, St Louis, USA). The promoter region of *nat* was identified using a BD GenomeWalker™ kit (Clontech, Takara, Japan). Four genomic DNA libraries were constructed by digesting the genomic DNA with four different restriction enzymes (DraI, EcoRV, PuvI and StuI). These libraries were ligated to GenomeWalker Adaptors. For the primary PCR, 1 μl of each library was subjected to Gene-Specific Primer 1 (NAT-GSP1) (Table 2), Adaptor Primer 1 (AP1) (provided with the kit) (Table 2) and Advantage 2 polymerase Mix (Clontech, Takara, Japan). The 25-fold-diluted primary PCR products were used as the template for the nested PCR with AP2 (provided with the kit) and NAT-GSP2 (Table 2). Secondary PCR products were cloned into pT7Blue, a TA cloning vector (Novagen, Darmstadt, Germany), by using ligation high Ver.2 (Toyobo, Osaka, Japan). Sequencing was performed using an ABI prism Big-Dye terminator cycle sequencing ready reaction mixture (PE Applied Biosystems, CA, USA) and a 3100xl Genetic Analyzer Sequencer (PE Applied Biosystems, CA, USA).

**Table 2:** List of primers used in the experiments. Underlined sequences are the T7 promotor.

Name of the primer	Sequence of the primer	Use	Size of the product (bp)
NAT-GSP1	GTTCATGGGTTCGTCGCGGAAGAAGAA	Promotor assay	–
NAT-GSP2	GCCTTTGTATCGTGTATGAGGGTTGCG	Promotor assay	–
AP1	GTAATACGACTCACTATAGGGC	Promotor assay	–
AP2	ACTATAGGGCACGCGTGGT	Promotor assay	–
pT7-F	ATGACCATGATTACGCCAAG	TA cloning	–
pT7-R	GTTTTCCCAGTCACGAC	TA cloning	–
NAT-T7-F	TAATACGACTCACTATAGG GAGATCGAAGTGATTGAAGAAGAGGA	dsRNA synthesis	585
NAT-T7-R	TAATACGACTCACTATAGG GAGACAGAACCCCTTAGTTTAGCG	dsRNA synthesis	
NAT-F	GCGTAATAAGGCCGTCAGAA	qRT-PCR	250
NAT-R	AGATTCACGTGGACATTTCAGC	qRT-PCR	
CYC-T7-F	TAATACGACTCACTATAGG GAGAATCAGATCGGCAGCACCAT	dsRNA synthesis	531
CYC-T7-R	TAATACGACTCACTATAGG GAGATCGTATTACGCGGTACCTACTT	dsRNA synthesis	
CYC-F	GTTACTGAACCGTAACACTCTCCAG	qRT-PCR	227
CYC-R	TCTCAGTATCCGGTTTCTCTTTGTA	qRT-PCR	
CLK-T7-F	TAATACGACTCACTATAGG GAGACGCAAAGTCTCTGAGCCAGT	dsRNA synthesis	511
CLK-T7-R	TAATACGACTCACTATAGG GAGAGGCAAGCGATTGCGTTTA	dsRNA synthesis	
CLK-F	TATGGAGAAAGATATGAAGCAGGAG	qRT-PCR	250
CLK-R	GTAGTACTAACCGTCTGGACAGTGG	qRT-PCR	
PER-T7-F	TAATACGACTCACTATAGG GAGATGTACTATACGCCAGTGACTGCTAC	dsRNA synthesis	407
PER-T7-R	TAATACGACTCACTATAGG GAGAGCACAGAGAGGAATCTGATGAATTT	dsRNA synthesis	
PER-F	AAGCCTATACGTCTCACTGAATCCT	qRT-PCR	219
PER-R	CTTTTCGTATACCGATGGTGTGTTA	qRT-PCR	
GFP-T7-F	TAATACGACTCACTATAGG GAGACCTGAAGTTCATCTGCACCAC	dsRNA synthesis	543
GFP-T7-R	TAATACGACTCACTATAGG GAGAACGAACTCCAGCAGGACCAT	dsRNA synthesis	
Actin-F	ACCAGAGAGGAAGTACTCTG	qRT-PCR	211
Actin-R	TTACAAAGCCTGAGTTGAGC	qRT-PCR	

### RNA Extraction and Synthesis of cDNA

The brain-suboesophageal complexes (BR-SOGs) of *A. pernyi* were dissected from pupae and immediately transferred to liq.N_2_. Total RNA was extracted from the BR-SOGs using RNAiso Plus reagent (Takara, Japan) following the instructions of the manufacturer. For construction of cDNA, total RNA was incubated at 70°C for 3 minutes, and then we used ReverTra Ace (Toyobo, Osaka, Japan) for cDNA construction following the instructions of the manufacturer.

### Preparation and Injection of dsRNA

Four groups of specific dsRNA were prepared by the same method. The first group was for *nat* (accession no. DQ372910) (dsRNA^NAT^); PCR product (585 bp) was prepared using gene-specific primers (NAT-T7-F and NAT-T7-R) (Table 2) in which T7 promoter was attached to the 5′ end of each primer with a gap of four nucleotides. The cDNA was used as a template for the PCR. The PCR product was purified with GFX PCR DNA and Gel Band purification kit (GE Healthcare, UK). The purified PCR product was used as a template for synthesizing dsRNA using MEGAscript RNAi kit (Ambion, CA, USA) according to the manufacturer’s instructions. The same protocol was used for generating the other three groups of the dsRNA, *cycle* (also known as *Bmal*) (accession no. AY330487) (dsRNA^CYC^), *clock* (accession no. AY330486) (dsRNA^CLK^) and *period* (accession no. U12769) (dsRNA^PER^) using gene-specific primers for each of them (CYC-T7-F, CYC-T7-R; CLK-T7-F, CLK-T7-R and PER-T7-F, PER-T7-R, respectively) (Table 2), and the size of the PCR products were 531 bp for *cyc*, 511 bp for *clk* and 407 bp for *per*. The control dsRNA was generated from a GFP gene of jellyfish (dsRNA^GFP^), which does not have any effect on the target gene [43]. Before injection of any of the four kinds of dsRNA, transfection reagent, Metafectene PRO (Biontex, Planegg, Germany), was mixed with dsRNA at a ratio of 1∶1 v:v. 1 μg of dsRNA was injected into the pupae for two successive days (total 2 μg dsRNA/pupa).

### qRT-PCR

RNAs and template cDNAs were prepared as mentioned above. qRT-PCR was performed by using SYBER Green Realtime PCR master mix (Toyobo, Osaka, Japan) and Applied Biosystem 7500 real PCR system (Applied Biosystems, CA, USA). Actin of *A. pernyi* (accession no. GU176616) was amplified with gene-specific primers (Actin-F, Actin-R) (Table 2) and quantified for C_T_ in each sample as an internal control. Each treatment was replicated three times. Quantitative analysis followed by a comparative C_T_ (ΔΔC_T_) method was used for analysis [44]. For each gene, the primers used in qRT-PCR were designed for the outer region of dsRNA, namely, primers for *nat* (NAT-F, NAT-R), *cyc* (CYC-F, CYC-R), *clk* (CLK-F, CLK-R) and *per* (PER-F, PER-R) (Table 2).

### Statistical Analysis

Data were expressed as the mean ± SEM. Significance of differences was determined using a one-way ANOVA followed by Fisher’s PLSD test using SPSS program (SPSS Statistic 17.0.1, 2008). All studies were performed by Sigma Plot 2004 version 9.01 (Systat Software).

## Results

### Colocalization of Circadian Clock Gene Products and Melatonin Synthesizing Pathway

Brains of *A. pernyi* were stained immunohistochemically, using anti-*Pa* PER, anti-*Ap* CYC, anti-*Ap* CLK, anti-*Bm* CYC, anti-*Dm* and *Ap* NAT, anti-rat HIOMT and anti-melatonin antibodies. Individual or adjacent sections of the same brain were compared after staining with combinations of two different antisera.

**Figure 1:**
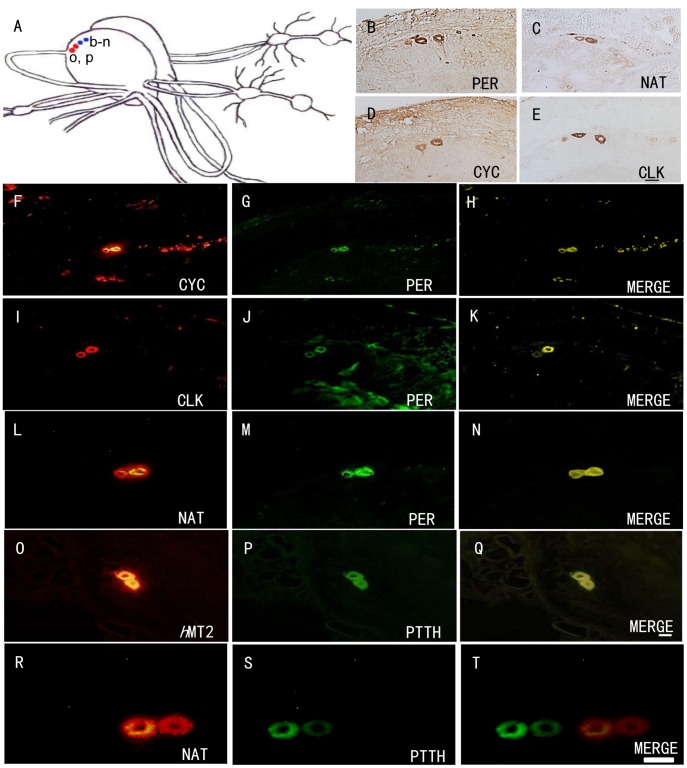
NAT-ir, PER-ir, CYC-ir and CLK-ir in the brain of *A. pernyi,* early pupal stage. (A) The topography of immunoreactive cells. Lower-case letters in the map indicate regions shown in the photographs (e.g., b is the site of B). (B) Two PER-ir neurons in the dorsolateral protocerebrum (DL). (C) Two NAT-ir neurons in the adjacent section to B in the DL. (D) CYC-ir in the DL region. (E) Two large CLK-ir neurons in the adjacent section to D in DL region. (F, I, L) CYC/CLK/NAT-ir in the DL region, respectively. (G, J, M) PER-ir in the same sections as (F, I, L) in the DL region, respectively. (H, K, N) Merged image of PER-ir and CYC/CLK/NAT-ir respectively in the DL region. (O) *h*MT2-ir in the DL regio. (P) PTTH-ir in the DL region. (Q) Merged image of *h*MT2- and PTTH-ir in the DL region. (L) MT-ir in the DL region. (M) PTTH- ir in the DL region. (N) Merged image of PTTH-ir and MT-ir in the DL region. Scale bars = 100 μm (B–E) and 100 μm (F–N).

A pair of neurosecretory cells showed PER/CYC/CLK-ir in the dorsolateral (DL) region of each hemisphere in the pupal brain (Fig. 1B, D, E, F–K). These results indicate that these cells are putative “clock neurons”. NAT-ir was also co-localized in the same neurons as PER-ir/CLK-ir/CYC-ir cells (Fig. 1L-N).

Melatonin-ir (MT-ir) and HIOMT-ir were also co-localized in the same “clock neurons” (Fig. S1). These results implicate that these “clock neurons” have a functional arylalkylamine metabolic pathway leading to melatonin.

### The PTTH-producing Neurons Express Melatonin Receptor 2 (*h*MT2)-ir

*h*MT2-ir and PTTH-ir were co-localized symmetrically in a pair of neurosecretory neurons in the DL (Fig. 1O–Q) that were juxtaposed to the PER/CYC/CLK/NAT-ir cells (Fig. 1R–T). This result suggests that the PTTH neurons possess melatonin receptor, which is a possible channel for circadian gating for PTTH release.

### Day/Night Fluctuation of Melatonin Content in the Head Ganglia

**Figure 2:**
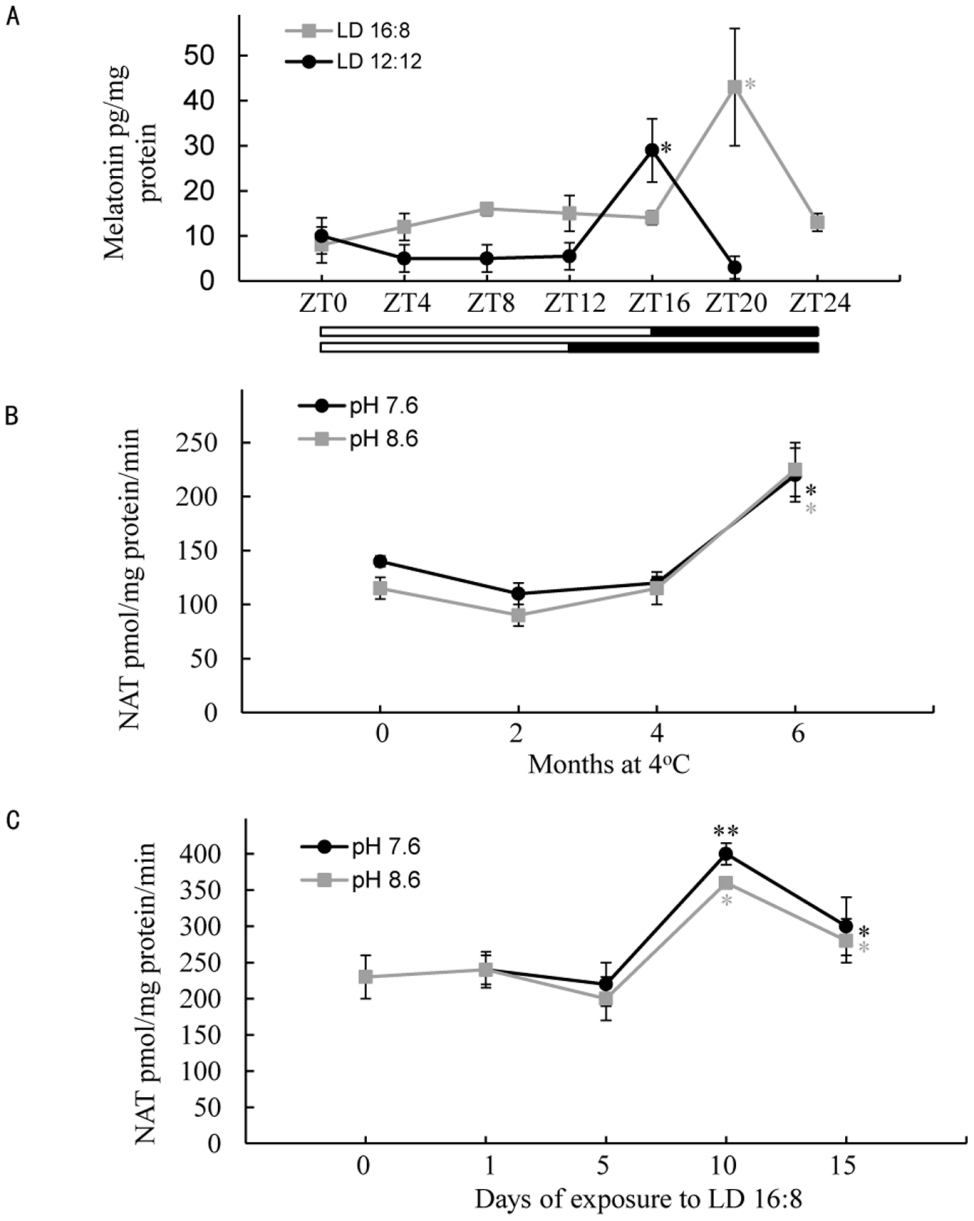
Changes in melatonin content and aaNAT activity in the brain-SOG in *A. pernyi*. Data are presented as mean ± SEM, n = 4–6 for each time point and treatment. A) Melatonin content at different Zeitgeiber times (ZTs), 2–3 days adults were kept under LD 16∶8 and 12∶12 at 25°C. Melatonin content was measured by RIA. B) Activity of aaNAT after diapause pupae were stored for designated months at 4°C. C) aaNAT activity after diapause pupae were kept for designated cycles of LD 16∶8 The activity was measured by radioenzymatic assay at pH 7.6 and 8.6. *P<0.05, **P<0.01.

Changes in melatonin content in the head ganglia from 2–3 days old adults maintained under LD 16∶8 were investigated by RIA. The results clearly showed a rhythmic fluctuation with a peak 4 hours after lights off. The peak level, 42.18 pg “melatonin”/mg protein, was significantly higher (p = 0.003) than the basal level of 12.64 pg/mg (Fig. 2A). Both the peak and the baseline levels were higher under LD 16∶8 than under 12∶12, a photoperiodic influence.

### Changes in NAT Activity with Diapause Termination

Radioenzymatic assay analysis showed that NAT activity in the head ganglia of the bivoltine strain of *A. pernyi* had two pH optima at 7.6 and 8.6. The activity of NAT from diapause pupae of this strain increased sharply between 5 and 10 days of exposure to LD 16∶8 following 4-months at 4°C, DD, which corresponded to a surge of ecdysteroid [45]. The activity also changed during cold storage. The level of activity in diapause pupae stored at 4°C, DD, for up to 4 months was as low as that of diapause pupae that were maintained under short-day conditions (LD 12∶12) at 25°C, but it gradually increased thereafter at both pH 7.6 and 8.6. (Fig. 2B, C).

### Analysis of Upstream Regulatory Region of *Ap nat* and Transcription Patterns in *nat*, *cyc* and *clk*

**Figure 3:**
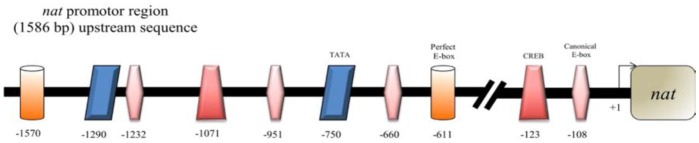
A diagramatic figure showing cis-elements in the promotor region of *nat*. Two perfect E-boxes (CACGTG) in orange, 4 canonical E-boxes (CANNTG) in pink, 2 CREs (NNNCGTCA) in red and 2 TATA motifs (TATAAA or TATAAAA) in blue were recognized.

**Figure 4:**
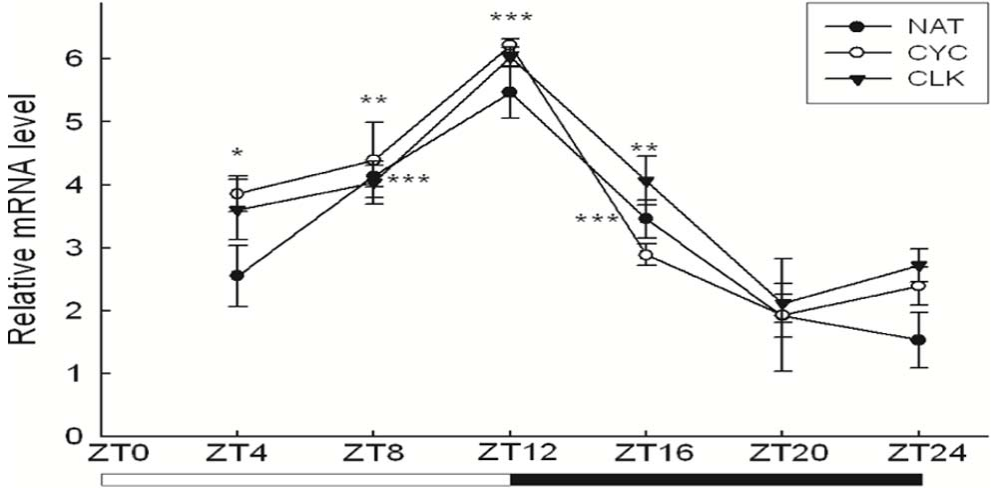
Real time PCR analyses of *Ap nat*, *Ap clk* and *Ap cyc* transcriptional levels. Transcriptional rhythms of the three genes have the same acrophase at Zt 12. *P<0.05, **P<0.01, ***P<0.001.

We cloned a cDNA encoding this NAT from *A. pernyi* (accession number DQ372910) and expressed the enzyme using a baculovirus expression system and confirmed the enzymatic activity [46]. We then investigated the upstream promoter region. The sequence is given in Fig. S2, as is a diagrammatic figure for the upstream sequence (Fig. 3) showing the sites of 2 perfect E-boxes (CACGTG) and 4 canonical E-boxes (CANNTG) as well as 2 CREs. To confirm the transcription regulation of *nat* by CLK/CYC we investigated the transcription patterns of *nat, clk and cyc* by real-time PCR. Transcription patterns of all three genes were rhythmic with the same phase relationship (Fig. 4).

### RNAi Against *nat* and the Effect on Photoperiodism

**Figure 5:**
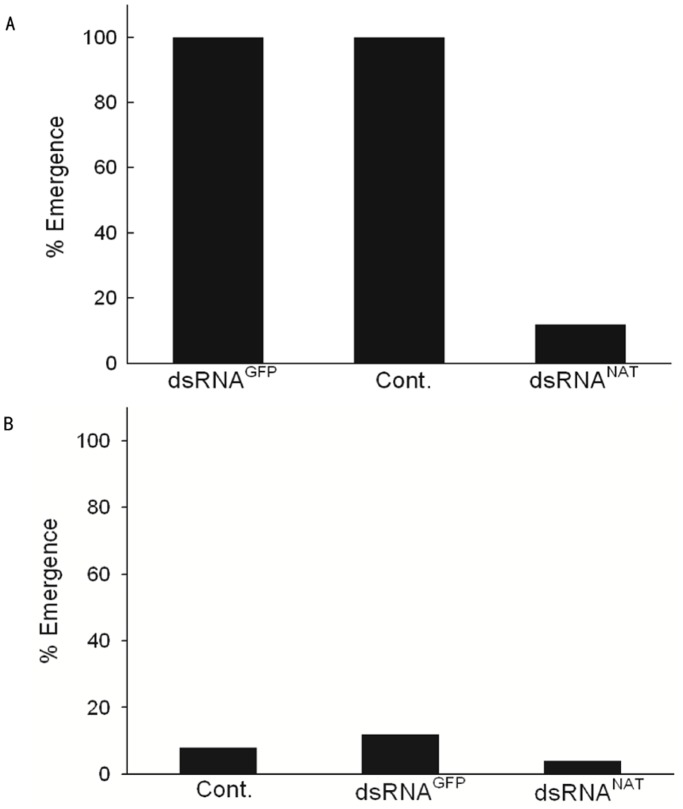
Photoperiodic terminations of diapause puape after RNAi against *nat*. dsRNAs against *Ap nat* (dsRNA^NAT^) or GFP (dsRNA^GFP^) were injected into the pupae. Nuclease-free water (NFW) is used as negative control (Cont.). The control and treated pupae were placed under LD 16∶8 or 12∶12. A) NFW- injected group and dsRNA^GFP^- injected group responded to long-day photoperiod producing moth emergence at 100% after 40 days under long-day condition but dsRNA^NAT^- injected group stayed in diapause for 40 days even under LD 16∶8. B) On the contrary, under LD 12∶12 all groups produced moth emergence not more than 20% after 40 days. n = 25–30 for each treatment.

After the injection of dsRNA^NAT^, diapause pupae were kept either under LD 16∶8 or LD 12∶12 at 25°C. *nat* expression level was reduced 48 hours after the injection of dsRNA^NAT^ (Fig. S3A). The pupae to which the dsRNA^NAT^ was applied failed to emerge even under LD 16∶8 (Figs 5A, S4A), while the control as well as dsRNA^NAT^-injected pupae stayed in diapause under LD 12∶12 (Figs 5B, S4B). Photoperiodism was intact upon the mock injection of control GFP dsRNA. Photoperiodism was thus impaired after RNAi mediated knockdown of *N*-actyltransferase. These results strongly suggest that *nat* is causally involved in photoperiodism.

### RNAi Against *cyc* and *clk*

**Figure 6:**
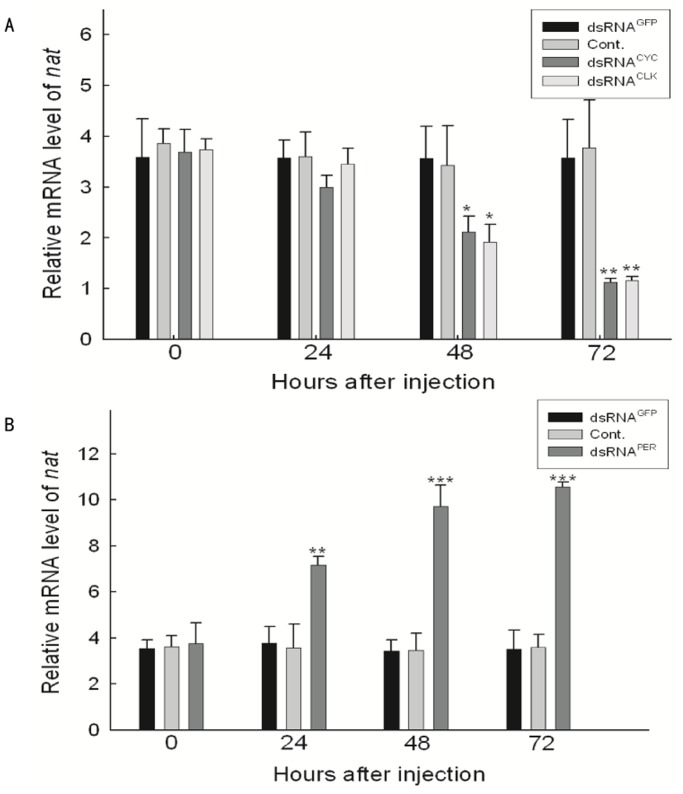
Effect of dsRNAs injections against some circadian clock genes on *nat* transcription. Data are presented as mean ± SEM, n = 5–8 for each time point and treatment. Pupae were maintained under LD 12∶12. A) dsRNAs of *cyc*, *clk*, and GFP were synthesized and injected to diapause pupae. NFW is used as negative control (Cont.). dsRNA^CYC^ and dsRNA^CLK^ suppressed *nat* transcription. B) dsRNA^PER^ up-regulated *nat* transcriptional levels after 24 hours from injection of dsRNA^PER^. *P<0.05, **P<0.01, ***P<0.001.

We then sought the link between *nat* regulation and the circadian clock using RNAi against two transcription modulators, CYC and CLK, which bind as heterodimers to E-box elements. The expression level of *cyc* declined significantly 48 hours after the injection of dsRNA^CYC^ (Fig. S3B), but the expression level of *clk* decreased only 24 hours after the injection of dsRNA^CLK^, (Fig. S3C). The expression level of *nat* was measured by real-time PCR. Fig. 6A shows that RNAi against either *cyc* or *clk* suppressed the expression level of *nat*. This result shows that *nat* is a clock-controlled gene (ccg) that is responsible for transmitting circadian output to an endocrine switch to release PTTH. Build-up of *nat* transcript may function as a condenser that is physiologically termed a photoperiodic counter [47].

### RNAi Against *per* and the Effect on Photoperiodism

**Figure 7:**
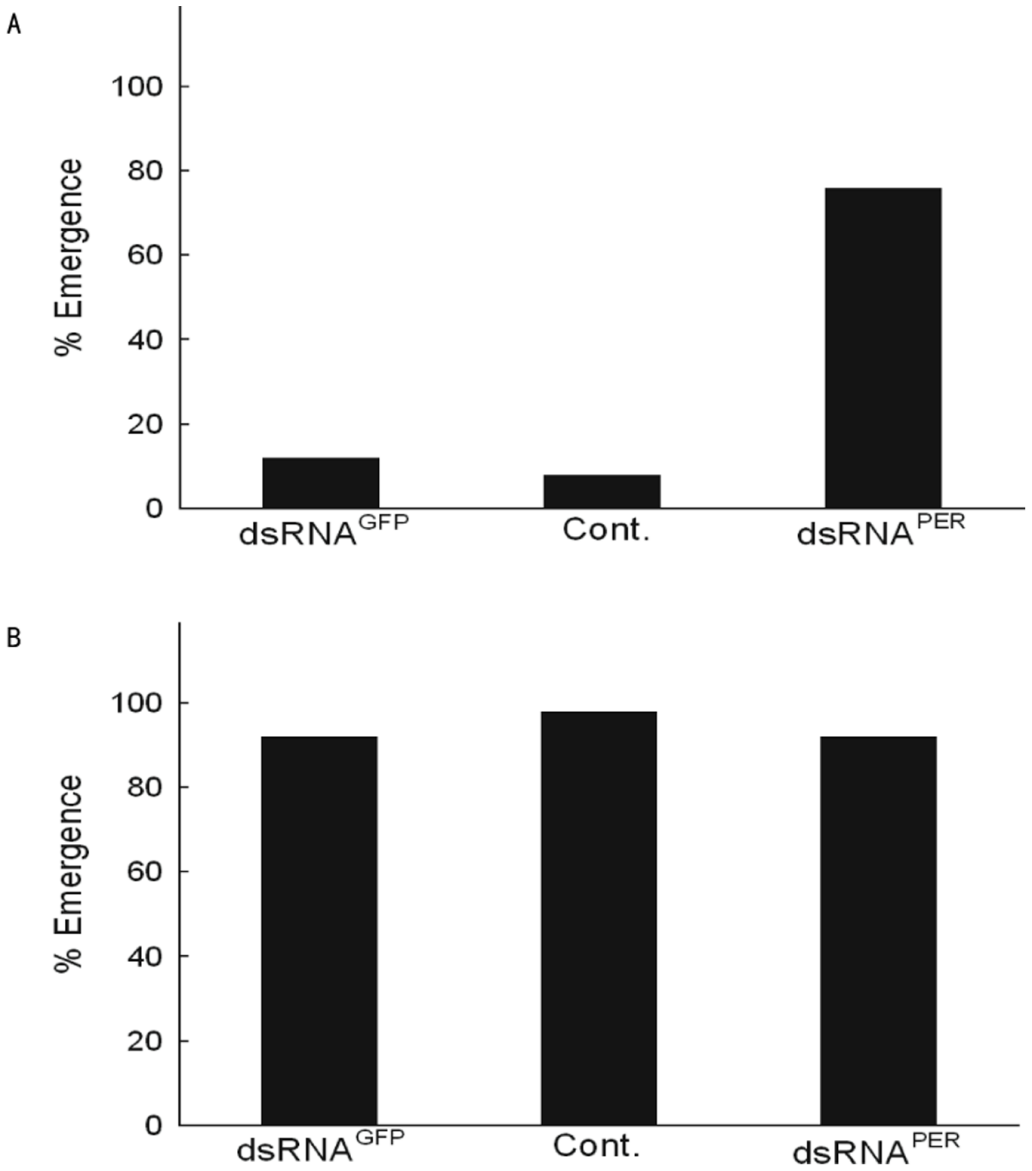
Effect of dsRNA against *per* on adult emergence under LD and SD. Pupae were injected with dsRNA^PER^, dsRNA^GFP^ and NFW (-negative control) (Cont.). n = 25–30 for each treatment. A) Diapause was terminated after injection of dsRNA^PER^ even under short-day (LD 12∶12). B) Injection of dsRNA^PER^ showed no difference from the controls under long-day (LD 16∶8).

To confirm that photoperiodism in *A. pernyi* operates based on the same basic framework of the circadian system in *D. melanogaster,* that is, a negative feed-back loop, we injected dsRNA against *per* (Fig. S3D) expecting it would upregulate *nat* transcription. Fig. 6B shows that this is indeed the case. RNAi against *per* inhibited the inhibition of transcription by PER, resulting in increased *nat* transcription. Pupae injected with dsRNA^PER^ showed a high proportion of emergence even under short-day conditions (L:D 12∶12) (Figs 7A, S4C). Meanwhile the dsRNA^PER^-injected pupae kept under L:D 16∶8 showed accelerated emergence compared with the control pupae (Figs 7B, S4D). This suggests that, in *A. pernyi*, photoperiodic time measurement is a function of the circadian system shared by different organisms such as *D. melanogaster* and rodents.

### “Melatonin” Content After Silencing *nat* and *per*

**Figure 8:**
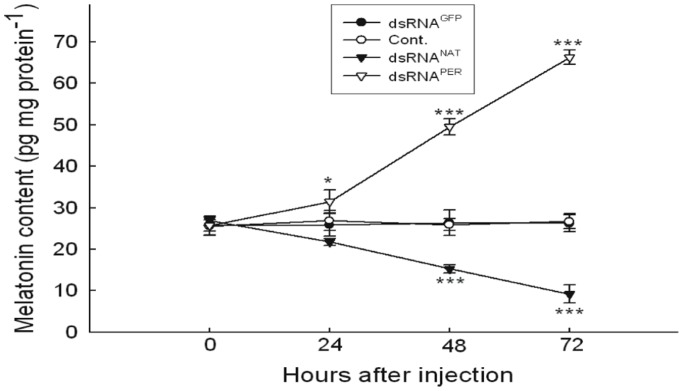
Effect of injection of dsRNAs against *nat* and *per* on melatonin level. Data are presented as mean ± SEM, n = 15 for each time point and treatment. Silencing *nat* decreased melatonin content at 48 hours after injection dsRNA^NAT^, while knocking down *per* upregulated melatonin level at 24 hours after dsRNA^PER^ injection. *P<0.05, ***P<0.001.

Using RIA, melatonin level was measured in the brain of diapause pupae injected with dsRNA^NAT^, dsRNA^PER^ and dsRNA^GFP^. The results showed a great decline 48 hours after the injection of dsRNA against *nat*. The results also showed that melatonin level was significantly higher after knocking down *per* by 24 hours. (Fig. 8).

## Discussion

Melatonin was previously identified as a hormone that affects melanophore concentration in frog tadpole [48]. Structurally, it is an indolamine, and among many vertabrates it is synthesized in the pineal organ, retina and gastrointestinal organs [49]. It is found in a wide range of organisms, including *Paramecium,* through higher plants, thus indicating that it has phylogenetically ancient origins. In early 1970s, it was found that the pineal organ contains circadian oscillations and that the organ synthesizes melatonin in a highly rhythmic manner due to an enzyme, arylalkylamine *N*-acetyltransferase (aaNAT). aaNAT is highly photosensitive and critical to photoentrainment in the melatonin rhythm; in some species, its levels fluctuate several hundred fold, in a circadian manner. Melatonin functions as an endocrine or biochemical token of uninterrupted darkness.

Although NAT sequences are not highly-conserved between vertebrates and invertebrates and roles for melatonin in invertebrate circadian rhythm regulation remain inconclusive, many lines of evidence suggest that, as seen in vertebrates, equivalent system also plays a critical role invertebrates. Melatonin has been identified by GC-mass analysis from locust compound eyes [50]. *B. mori*, which is taxonomically closely related to *A. pernyi*, has also shown a circadian rhythm for melatonin levels and has enzymatic activities of NAT and HIOMT [51]. Also, melatonin intake synchronized a locomotor rhythm in the house cricket, *Acheta domestica* [52].

PER-, CYC-, CLK-, HIOMT-, melatonin- and NAT-ir were found to be coexpressed in the dorsolateral neurosecretory cells. This suggests that these neurons function as circadian clock neurons. Importantly, the most external set of these putative clock neurons in the dorsal region of the protocerebrum in *A. pernyi* are juxtaposed to PTTH-secreting cells that expressed MT-ir. Takeda et al., [1] have shown rhythmic fluctuations of melatonin content in the BR-SOG of *A. pernyi* by RIA under LD cycles. These results strongly suggest that melatonin is the output messenger of the circadian clock cells in *A. pernyi.*

An approximate 4–6 hour lag was observed between *per* mRNA and PER protein expression in *D. melanogaster* [53]. This time lag is due to the combination of accumulation of mRNA and degradation of protein. The present study may show that *A. pernyi* has a somewhat unique biological clock system different from that of *D. melanogaster.* PER of *A. pernyi,* as in *P. americana,* does not exhibit massive migration to the nucleus, even though a trace amount of PER does make a nuclear translocation [54] and the nuclear localization signal (NLS) is present, although its sequence is less conserved between *Drosophila* and *Antheraea* than between *Drosophila* and *Periplaneta*.

Pigment dispersing factor (PDF), thought to be the output messenger of the biological clock in *D. melanogaster* [18] is not co-localized in PER-expressing cells in *A. pernyi* [2]. These results suggest that the clock cells entrain a melatonin rhythm, but not PDF in *A. pernyi*, and melatonin may signal the endocrine effector, PTTH. This notion is supported by the fact that repeated injection of melatonin *in vivo* terminated diapause [55] **.** As Richiter et al., [56] have reported, melatonin stimulates PTTH secretion in an *in vitro* system in *P. americana*, and inhibited it. This effect was blocked by luzindole, a melatonin receptor antagonist.

In addition to the photoperiodic clock itself, photoperiodism requires a counter, or accumulator, in order for a threshold of the number of effector signals to be reached to execute a hormonal switch in a developmental program. The “melatonin” synthesis or transcript accumulation of *nat* could serve as this critical information.

The presence of MT-ir on PTTH cells also suggests that melatonin could release or gate the release of PTTH. Photoperiodism has long been speculated as a function of circadian systems and it is this subject that the “father of chronobiology”, Colin S. Pittendrigh, examined in his last study [57], however the molecular nature of this function remains unresolved. The current results align nearly all of the functional subunits comprising the photoperiodic system along the indoleamine pathway in *A. pernyi*; with the exception of the photoreceptor, this includes the circadian clock, photoperiodic counter and the endocrine switch mechanism.

It must be noted that two serotonin (5HT) receptors have been found in the PTTH neurons in this species. Although “melatonin” may trigger the release of PTTH, serotonin interferes with this release in order to more strictly regulate the process of diapause [58]. This binary mechanism likely serves for the stability in photoperiodic system. NAT can balance the ratio of melatonin and 5HT. 5HTR_B_ levels have been shown to be regulated by photoperiod and dsRNA^5HTRB^ injection stimulated diapause termination even under shortened day conditions. Also 5HT injection intensified diapause and a 5HT depletion compound, 5,7 dihydroxytryptamine, terminated it [58].

NAT activity was shown to gradually increase during diapause termination at a low temperature and sharply increased when exposed to a long day [1]. A parallel increase in “melatonin” content in the brain is expected no matter whether the synthesized “melatonin” is directly released or accumulated until the gated release with a suitable quantity. Activation of *nat* may be affected not only via photoperiodic conditions but also via neurotransmitters, as in mammals, low temperature or other stresses. Mechanical shaking can terminate pupal diapause of *A. yamamai,* a closely related species of *A. pernyi.* Analyses of the *nat* promoter revealed CRE elements, in addition to the E-boxes. PTTH release may thus be regulated by multichannel mechanisms, though further analyses are required in this regard.

Direct evidence that *nat* is the cogwheel or critical gear of photoperiodism came from RNAi experiments. Direct engagement of circadian transcription modulator CYC/CLK in *nat* transcription was demonstrated by RNAi targeting of *cyc* and *clk.* This claim is further supported by the presence of multiple E-boxes in the promoter region of *nat*.

dsRNA against *per* also demonstrated that this photoperiodic time measurement is based on a very common circadian system shared by many different organisms such as fruit fly, rodents and humans, namely a negative feedback loop. Indeed, this is a very simple facet of the circadian clock system and one of its key functions is indolalkylamine metabolism. It is also evolutionarily very reasonable because melatonin is involved in many light-mediated physiological mechanisms such as pigmentation, photoreception, circadian rhythms, sleep and ROX scavenging in a wide range of organisms including *Paramecium* as well as higher plants. These are collectively regarded as UV adaptations and have an old evolutionary origin.

Disruption of photoperiodism with RNAi against circadian clock genes has been documented in several cases in insects, for example, the larval photoperiodic diapause of the drosophilid fly *Chymomyza costata* [59] and the regulation of nymphal development in the cricket *Modicogryllus siamensis* [60]. In the case of *C. costata*, RNAi against *tim* forced the insect to express a non-diapause phenotype, even under diapause-inducing short-day conditions [59]. In the second case, RNAi against *per* produced arrhythmic locomotor activity under light-dark conditions as well as constant darkness [60]. In the bean bug, *Riptortus clavatus*, RNAi of some circadian clock genes disrupted photoperiodic responses in females: RNAi targeting *per* and *cry-m* influenced the ovarian development even under diapause-inducing short-day conditions, but RNAi against *cyc* suppressed ovarian development even under non-diapause-inducing long-day conditions [61], [62]. RNAi against *clk* is similar to *cyc* RNAi, not only abolishing the cuticle deposition rhythm but also photoperiodic response. However, these studies have not clarified how circadian system affects photoperiodism.

Diapause initiation/maintenance and termination are two distinct phenotypic expressions and are mutually exclusive. Our proposed model based on the first phenotypic expression being executed by 5HT and the second by melatonin can be explained with a simple assumption that *nat* is the critical gene that regulates 1) relative amounts of seratonin and melatonin, 2) the circadian influence on photoperiodism and 3) a discrete switch for two exclusive phenotypic expressions. To the best of our knowledge the current study provideds first evidence for a molecular system-regulating pathway from the clock to endocrine switch of insect photoperiodism in a step-by-step fashion.

## Conclusion

The molecular mechanisms of photoperiodism, considered as a function of circadian system, remain an unsolved mystery. Through employing RNAi, immunohistochemistry, RIA and radioenzymatic assay to a classical system of insect endocrinology, *Antheraea pernyi,* we demonstrated that arylalkylamine *N-*acetyltransferase (aaNAT) is the critical conjunct between the circadian system to the endocrine switch releasing or blocking the release of prothoracicotropic hormone (PTTH), and avoiding/terminating or initiating/maintaining pupal diapause, respectively. dsRNA^NAT^ disrupted photoperiodic effect, dsRNA^CLK or CLC^ suppressed NAT transcription, which showed that: the *aaNAT* is a circadain-controlled gene, and the circadian transcription factor CLK/CYC heterodimer binds to E-boxes in the upstream regulatory region of *nat*. RNAi against PER, a repressor to this binding released the inhibition, significantly amplifying *aanat* transcription and resulting in early diapause termination. The PTTH neurons have been shown to co-express a melatonin receptor (MT)- and two 5HTRs-ir. Repeated injections of melatonin stimulated early diapause termination and the injection of 5HT correlated with diapause continuation, and 5HT and melatonin antagonist injections resulted in the opposite effect. Melatonin, NAT activity, CYC, CLK and NAT transcription all fluctuated in circadian manner and NAT can change the 5HT/melatonin balance in an antagonistic manner. This parsimoniously explains all basic elements underlying photoperiodism.

## Supporting Information

Figure S1 **Immunohistochemical reactivities to antisera against (A) melatonin, (B)** ***Dm*** **aaNAT, (C) HIOMT, and (D)** ***Dm*** **aaNAT in adjacent 8 μm sections (A/B and C/D).**
(TIF)

Figure S2 **The nucleotide sequence of the promoter region for** ***A. pernyi nat.*** Red highlighted sequences are perfect E-boxes, brown are CRE, Pink are canonical E-boxes, blue are TATA-motif and the green highlighted sequence is the start codon of *nat.*
(TIF)

Figure S3 **Effect of dsRNA injections on mRNA levels.** Diapause pupae were injected and kept under LD 12∶12 at 25°C after dsRNA injections. Data are presented as mean ± SEM, n = 5–8 for each time point and treatment. A) mRNA^nat^ after injections of dsRNA^GFP^ and dsRNA^NAT^. B) mRNA^cyc^ after injections of dsRNA^GFP^ and dsRNA^CYC^. C) mRNA^per^ after injections of dsRNA^GFP^ and dsRNA^PER^. D) mRNA^clk^ after injections of dsRNA^GFP^ and dsRNA^CLK^. NFW is used as negative control (Cont.). *P<0.05, **P<0.01, ***P<0.001.
(TIF)

Figure S4 **Adult emergence after injection of dsRNA^NAT^, and dsRNA^PER^. Effect of dsRNA^NAT^ on adult emergence under LD and SD (A, B, respectively).** dsRNA^PER^ effect on adult emergence after keeping the diapause pupae under SD and LD (C, D, respectively). n = 25–30 for each treatment.
(TIF)
