# Correction: *N-acetyltransferase (nat)* Is a Critical Conjunct of Photoperiodism between the Circadian System and Endocrine Axis in *Antheraea pernyi*

**DOI:** 10.1371/journal.pone.0235916

**Published:** 2020-07-02

**Authors:** The PLOS ONE Editors

Following publication of this article [1], questions were raised regarding the reporting of methodological and reagent information and about the description of the antibodies used in this study. Upon follow up with the authors, this notice provides additional information about the antibodies used in this study.

Anti-rat liver HIOMT (monoclonal) was a gift from the late Dr. Takeo Deguchi; no further information or references are available about this antibody.

Anti-A*p*PTTH serum raised in rabbit was a gift from Dr. Ivo Sauman. Additional information about the specificity and production of the anti-A*p*PTTH antibody can be found in [2].

References 5 and 40 of the original article are incorrectly cited in the section “Production of Other Antibodies and Anti-peptide Antibodies” and do not provide information about the anti-melatonin and anti-*h*MT2 sera. The product details of one of the anti-melatonin antibodies used for immunohistochemical staining (IHC) are not available at the time of publication of this notice. A second anti-melatonin antibody purchased from Stockgrand Ltd, product number AB/S/01, previously HP/S/704-6483 (also used for the Radioimmunoassay (RIA) procedure) had weaker reactivity in IHC. The anti-*h*MT2 sera was purchased from Santa Cruz, (MEL-1B-R antibody H-18) primary antibody of human origin (Santa Cruz Biotechnology, Inc., US; sc-13174).

The manufacturer and host species of the secondary antibodies used in immunohistochemical staining are specified in the Methods section, but the product numbers are not available.

As described in the Methods section, the authors produced antisera against *Pa* (*Periplaneta americana*) PER, *Ap (A*. *pernyi)* CYC, *Ap* CLK, *Dm (Drosophila melanogaster)* aaNAT and *Ap* aaNAT, *Bm (Bombyx mori)* CYC and *Bm* CLK. Dilutions used are provided in the article. Specificity of these antisera was validated by controls with the omission of the primary antibody and preabsorption tests.
