# Supplementary figures and images for: N-acetyltransferase (nat) Is a Critical Conjunct of Photoperiodism between the Circadian System and Endocrine Axis in Antheraea pernyi
Source: PLoS One. 2014 Mar 25;9(3):e92680. doi: 10.1371/journal.pone.0092680 (PMC3965458; doi:10.1371/journal.pone.0092680)

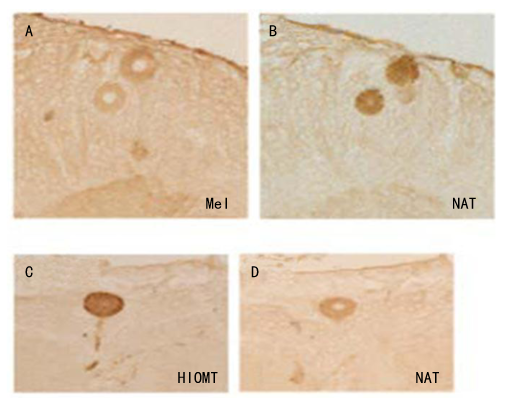

Supplement: Figure S1 — Immunohistochemical reactivities to antisera against (A) melatonin, (B) Dm aaNAT, (C) HIOMT, and (D) Dm aaNAT in adjacent 8 μm sections (A/B and C/D). (TIF) [file pone.0092680.s001.tif]

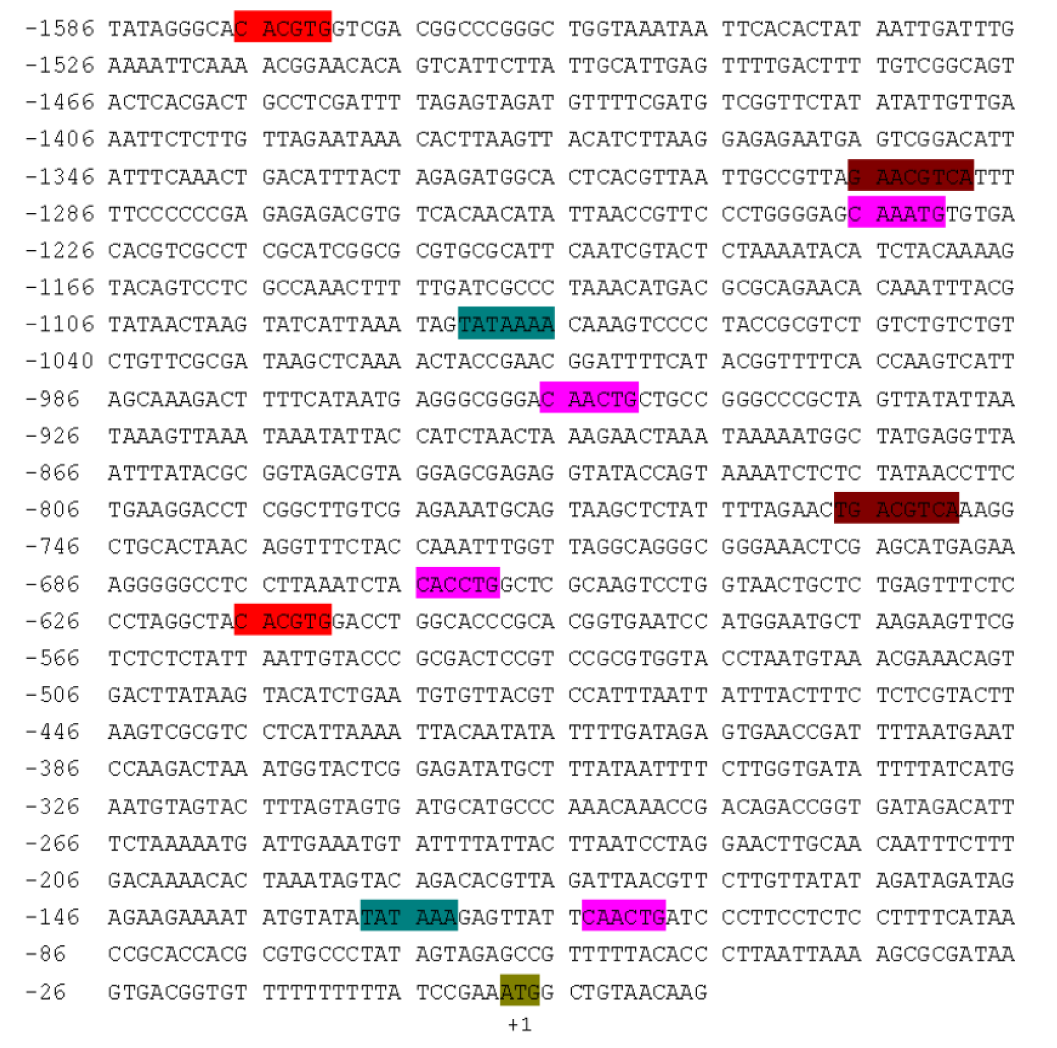

Supplement: Figure S2 — The nucleotide sequence of the promoter region for A. pernyi nat. Red highlighted sequences are perfect E-boxes, brown are CRE, Pink are canonical E-boxes, blue are TATA-motif and the green highlighted sequence is the start codon of nat. (TIF) [file pone.0092680.s002.tif]

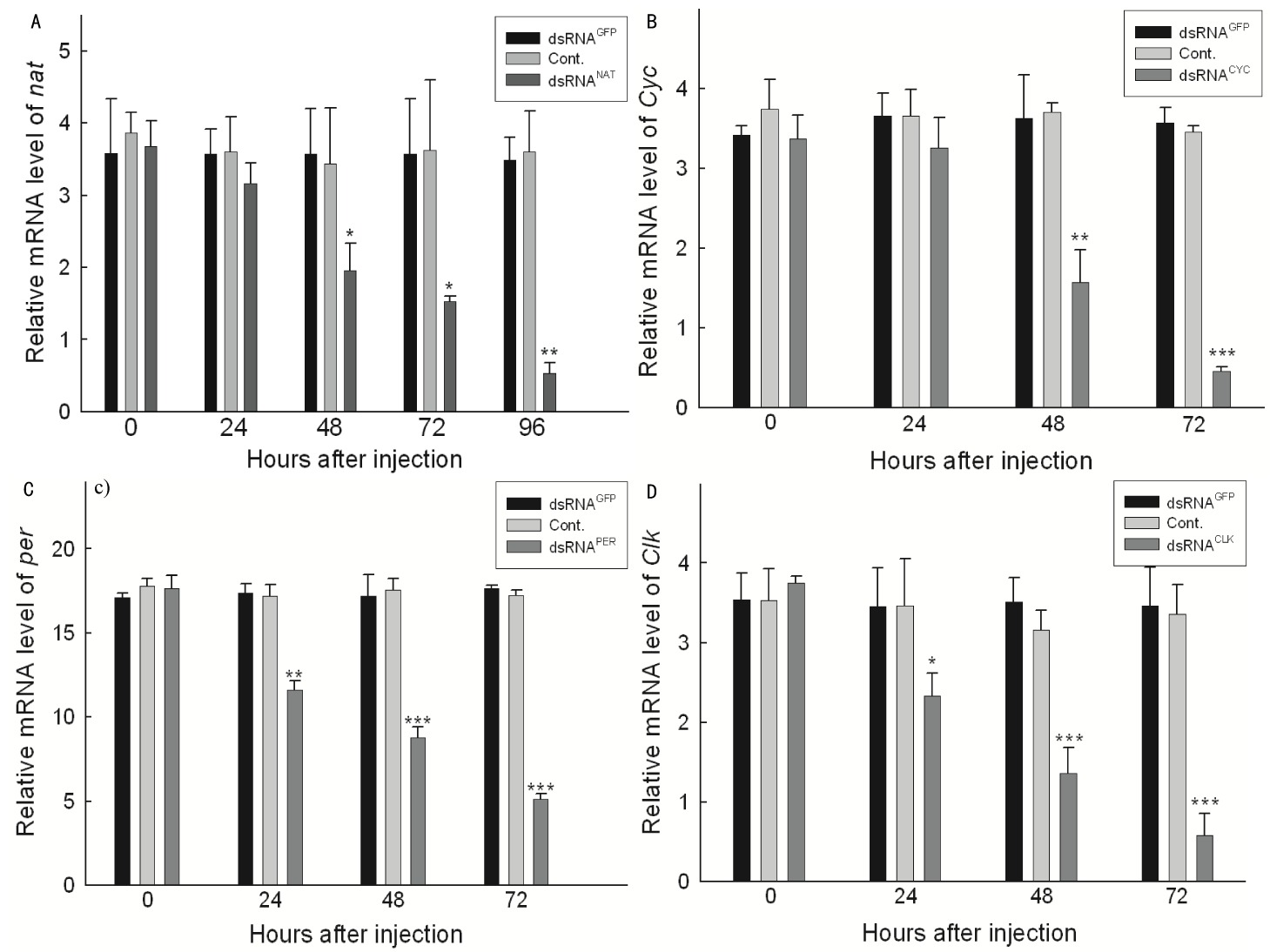

Supplement: Figure S3 — Effect of dsRNA injections on mRNA levels. Diapause pupae were injected and kept under LD 12∶12 at 25°C after dsRNA injections. Data are presented as mean ± SEM, n = 5–8 for each time point and treatment. A) mRNAnat after injections of dsRNAGFP and dsRNANAT. B) mRNAcyc after injections of dsRNAGFP and dsRNACYC. C) mRNAper after injections of dsRNAGFP and dsRNAPER. D) mRNAclk after injections of dsRNAGFP and dsRNACLK. NFW is used as negative control (Cont.). *P<0.05, **P<0.01, ***P<0.001. (TIF) [file pone.0092680.s003.tif]

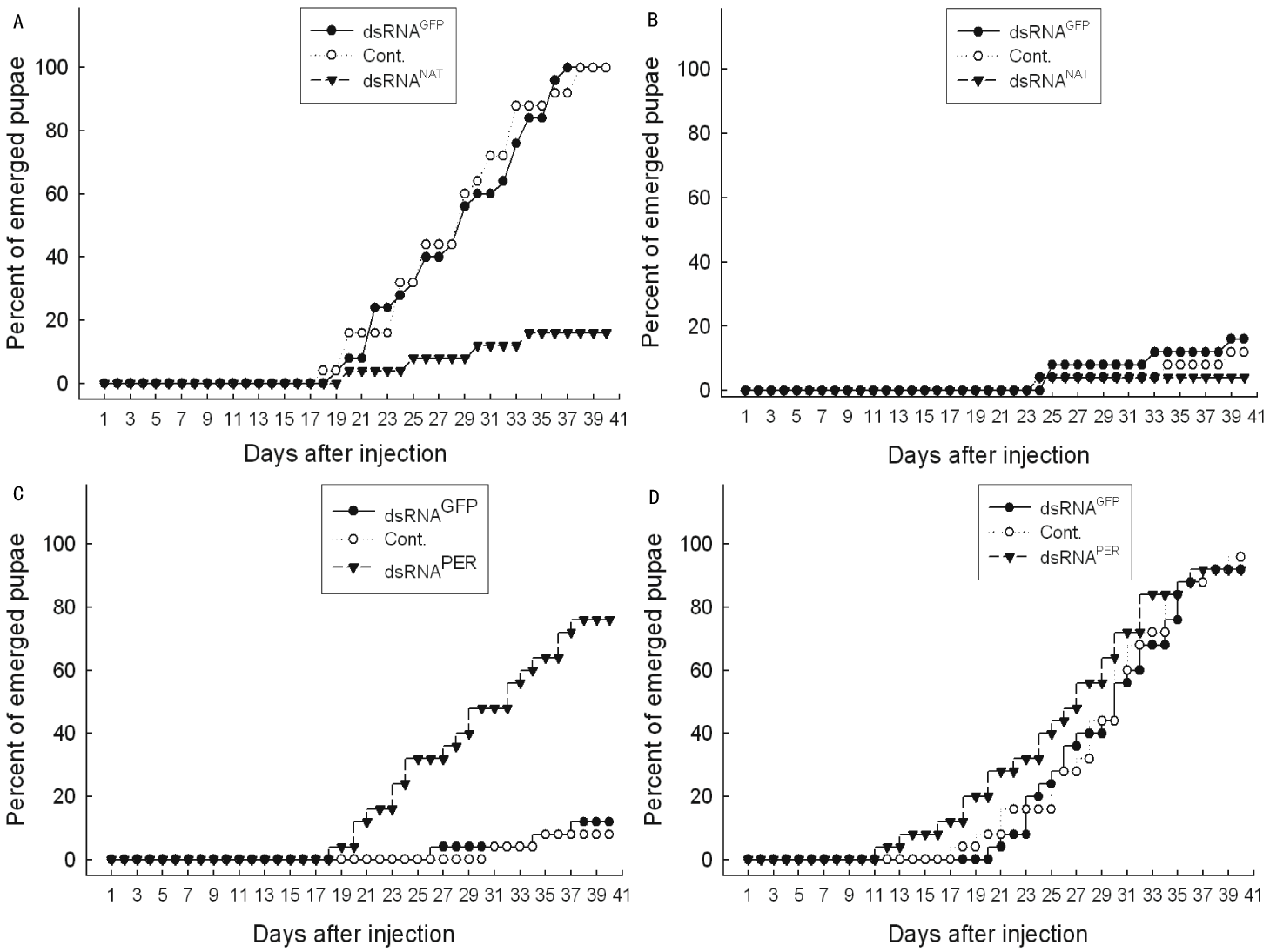

Supplement: Figure S4 — Adult emergence after injection of dsRNANAT, and dsRNAPER. Effect of dsRNANAT on adult emergence under LD and SD (A, B, respectively). dsRNAPER effect on adult emergence after keeping the diapause pupae under SD and LD (C, D, respectively). n = 25–30 for each treatment. (TIF) [file pone.0092680.s004.tif]
